# Wine Microbiology and Predictive Microbiology: A Short Overview on Application, and Perspectives

**DOI:** 10.3390/microorganisms10020421

**Published:** 2022-02-11

**Authors:** Leonardo Petruzzi, Daniela Campaniello, Maria Rosaria Corbo, Barbara Speranza, Clelia Altieri, Milena Sinigaglia, Antonio Bevilacqua

**Affiliations:** Department of Agriculture, Food, Natural Resources and Engineering (DAFNE), University of Foggia, 71122 Foggia, Italy; leonardo.petruzzi@unifg.it (L.P.); daniela.campaniello@unifg.it (D.C.); mariarosaria.corbo@unifg.it (M.R.C.); barbara.speranza@unifg.it (B.S.); clelia.altieri@unifg.it (C.A.)

**Keywords:** predictive microbiology, modelling, experimental design, wine, oenology

## Abstract

Predictive microbiology (PM) is an essential element in food microbiology; its aims are the determination of the responses of a given microorganism combining mathematical models with experimental data under certain environmental conditions, and the simulation a priori of the growth/inactivation of a population based on the known traits of a food matrix. Today, a great variety of models exist to describe the behaviour of several pathogenic and spoilage microorganisms in foods. In winemaking, many mathematical models have been used for monitoring yeast growth in alcoholic fermentation as well as to predict the risk of contamination of grapes and grape products by mycotoxin producing fungi over the last years, but the potentialities of PM in wine microbiology are underestimated. Thus, the goals of this review are to show some applications and perspectives in the following fields: (1) kinetics of alcoholic and malolactic fermentation; (2) models and approaches for yeasts and bacteria growth/inactivation; (3) toxin production and removal.

## 1. Introduction: Predictive Microbiology

Predictive microbiology (PM) is often considered to have its origins in the early 20th century, when Bigelow et al. proposed a log-linear model describing bacterial death kinetics after thermal processing. Nowadays, about 100 years later, these results are still applied by the food industry [1]. In PM, mathematical models are used to predict microbial behaviour, as affected by different environmental conditions [2], and there are several ways to classify models, depending on the event (growth or inactivation), theoretical foundation (empirical models or mechanistic functions), dependent variables, and input conditions (primary, secondary, and tertiary models) [3]. A possible classification of the models using the growth rates could be as follows [2]:

1. Kinetic models using the growth rate as the dependent variables, to focus either on growth or inactivation;
2. Probability models, generally based on the logit approach (probability to grow or die in some defined conditions);
3. Empirical models. They are first- or second-order polynomial equations, describing data with mathematical relationships (curve fitting) [3]. The main disadvantage of such models is the accuracy of the prediction that might drop severely with an extrapolation approach [2];
4. Mechanistic or deterministic models. They have a robust theoretical background and could contribute to understanding and exploring the phenomenon beyond a biological process [3].

Another classification of models in PM is the traditional three-level scheme, which is based on the dependent variable (primary vs. secondary models) or the aims (interpolation or prediction; primary/secondary models vs. tertiary models):

- Primary models focus on microbial evolution over time [2]. They include death models, D values (or thermal inactivation), inactivation/survival models, growth rate values, and even estimation of lag time, based on the assumption that microbial growth can be described by a sigmoid (Baranyi, Gompertz, Huang, and logistic models) [1].
- The secondary models describe the influence of environmental conditions (pH, temperature, a_w_, nutrients, antimicrobial compounds, etc.) on the kinetic parameters evaluated through primary models (growth rate, lag phase, inactivation rate, etc.) [2]. Surface response, Arrhenius, square root, and Bĕlehrádek models based on the gamma concept are some examples of secondary models [1].
- Finally, the tertiary models are computer tools used to simulate a priori microbial growth [2].

PM is extensively used in food science and technology, and many models have undergone a robust and appropriate validation, but there are still some limitations or drawbacks due to the complex behaviour of microorganisms in food systems [2]. Some models are designed and developed only for a well-defined range of variables or in laboratory systems; therefore, they could not be used in real food matrices, because some factors are not assessed (variability in microbial population, stress and stress adaptation, and physicochemical properties of the food structure) [4].

Another limitation of some PM tools is that they do not consider the depletion of nutrients and the accumulation of by-products in the medium. This limit was described in the past by Van Impe et al. [5], who proposed the so-called *S/P* models. These models are based on a simple theoretical background: Microbial growth could be strongly affected by the consumption of a limiting substrate (*S*) and/or the production of a metabolic waste (*P*). The global structure of these models, called *S/P* equations, reads as follows:

(1) $$\frac{dN}{dt}=\mu_{Q}\left(Q\right)\cdot \mu_{max}\;\cdot \mu_{S}\;\left(S\right)\cdot \;\mu_{p\;}\left(P\right)\cdot N$$

In this equation, *N* is the cell number; the term (*μ_Q_*(*Q*)) describes the lag phase, while the exponential phase is described by the second factor (*μ_max_*). The new factors related to the consumption of a limiting substrate and the production of the metabolic waste are described by *μ_S_*(*S*) and *μ_p_*(*P*). *μ_S_*(*S*) and *μ_p_*(*P*) are two equations, related to the Monod constant:

(2) $$\mu_{S}\;\left(S\right)=\frac{S}{S+K_{S}}\;\mu_{p\;}=1-\frac{P}{K_{p}}$$

where *K_S_* is a constant, called substrate saturation constant or substrate affinity constant, and *K_p_* is the inhibition constant.

These models can be particularly useful to describe alcoholic fermentation, where the metabolic performances of yeasts rely upon the consumption of sugar (*S*) and the production of ethanol (*P*).

## 2. Alcoholic Fermentation

Alcoholic fermentation involves many variables (both process’ variables and yeasts), such as the variety of must, microbiota, and winemaking technology. Process’ variables are often controlled empirically [6], while yeast growth is checked either directly through plate count and molecular methods or indirectly through chemical parameters such as CO_2_ emission, alcohol production, sugar consumption [7]. These approaches could be time consuming or expensive, while a predictive model could be useful to know a priori the process.

In this context, in the literature, there are several kinetic models for early diagnosis of stuck or sluggish winemaking processes [8]. A first physical and mathematical model was proposed by Coleman et al. [9]. This model consists of the following five coupled equations:

(3) $$\frac{dX}{dt}=\mu X_{A}\;\frac{dX_{A}}{dt}=\mu X_{A}-k_{d}X_{A}\;\frac{dN}{dt}=-\frac{\mu X_{A}}{Y_{X/N}}\;\frac{dE}{dt}=\beta X_{A}\;\frac{dS}{dt}=-\frac{\beta X_{A}}{Y_{E/S}}$$

where the five state variables are total (*X* (g/L)), and active biomass (*X_A_* (g/L)), nitrogen (*N* (mg/L)), ethanol (*E* (g/L)), and sugar (*S* (g/L)); *k_d_* is the death rate or rate of cell inactivation; *Y_X/N_* is a yield coefficient of biomass on nitrogen; *Y_E/S_* is the stoichiometric coefficient describing the formation of ethanol from sugar.

This model could accurately predict fermentation as a function of temperatures and initial conditions, but it has a main limit because it cannot accurately predict cell mass after the exponential growth phase, nor can it predict nitrogen utilisation by yeast.

An important contribution to the application of PM on wine was given by Assar et al. [8], who made some models in polynomial forms available in the literature, such as the functions of Pizarro and Scaglia, respectively.

The Pizarro model is defined as follows:

(4) $$\frac{dX}{dt}=\mu \cdot X\;\frac{dN}{dt}=-V_{N}\cdot X\;\frac{d\left[\mathrm{EtOH}\right]}{dt}=V_{\mathrm{EtOH}}\cdot X\;\frac{dS}{dt}=-V_{S}\cdot X\;\frac{d\left[\mathrm{Gly}\right]}{dt}=V_{\mathrm{Gly}}\cdot X$$

The Scaglia model is defined as follows:

(5) $$\frac{dX}{dt}=\left(F_{\mu }\cdot \mu -F_{\tau }\cdot \tau \right)\cdot X-F_{\mu }\cdot \beta \cdot X^{2}\;\frac{d\left[\mathrm{EtOH}\right]}{dt}=\frac{1}{Y_{{\mathrm{CO}}_{2}/\mathrm{EtOH}}}\cdot \frac{d{\mathrm{CO}}_{2}}{dt}\;\frac{dS}{dt}=\left(\left(V_{S}+V_{S_{0}}\right)\cdot X-\frac{0.00002}{Y_{X/S}}\cdot X^{2}\right)\;\frac{d{\mathrm{CO}}_{2}}{dt}=V_{{\mathrm{CO}}_{2}}\cdot X+\frac{d\left({\mathrm{CO}}_{2}Form\right)}{dt}\;\frac{dX}{dt}=\mu \cdot X\cdot \left(1-\frac{\beta }{\mu }\cdot X\right)$$

Generally, these models include some main state variables, such as biomass (*X*), ethanol (EtOH), nitrogen (*N*), sugar (*S*), glycerol (Gly), and carbon dioxide (CO_2_). Moreover, the functions consider also some coefficients deriving from flux analysis (terms with the symbol *V*), growth or decay rates (*μ*), yield coefficients (*Y*), correction (*F*), or regression coefficients (*β*).

Each model has benefits and limits, depending on the method for solving the equations; for example, the best regression was found for sugar consumption, especially at high temperatures. In addition, Coleman and Scaglia models worked better in transient phases, while the Pizarro function was more robust in steady-state conditions.

Del Nobile et al. [10] proposed a deterministic model to predict the growth curve of microorganisms from inoculation to death. The model was built at two different temperatures and nitrogen concentrations, and the starting point was that growth is affected by nitrogen, while ethanol amount influences cell death.

Cell concentration can be described as follows, with *R*_1_ and *R*_2_ two functions describing proliferation and death rates, respectively:

(6) $$\frac{dP\left(t\right)}{dt}=R_{1}\left(t\right)-R_{2}\left(t\right)\;\;$$

The model is completed by a set of three equations, describing nitrogen (*N*) and sugar consumption (*S*) and ethanol production (*E*) (in the first equation, *Y_X/N_* is the yield of conversion of nitrogen into biomass, while in the third function, *Y_E/S_* is the stoichiometric coefficient for sugar conversion into ethanol).

(7) $$\frac{dN\left(t\right)}{dt}=\frac{\mu_{0}\cdot \left\{\exp\left\{{\left[N\left(t\right)\right]}^{n_{c}}\right\}-1\right\}\cdot P\left(t\right)}{Y_{X/N}}\;\frac{dE\left(t\right)}{dt}=\left[\beta_{0}\cdot S\left(t\right)\right]\cdot P\left(t\right)\;\frac{dS\left(t\right)}{dt}=-\frac{\left[\beta_{0}\cdot S\left(t\right)\right]\cdot P\left(t\right)}{Y_{E/S}}$$

Apart from model development, another goal for PM microbiology in wine is the evaluation of the goodness of some existing models to interpolate experimental data and gain a robust estimation of the most important kinetic parameters (lag phase, and growth rate). Therefore, DeNittis et al. [7] performed an experiment with 12 yeast strains (7 belonging to *Saccharomyces sensu stricto* group, 1 to *Saccharomyces cherensiensis*, 1 to *Saccharomyces beticus*, and 3 to a non-*Saccharomyces* group) and evaluated the growth kinetic through Biolog. Data were fitted through Gompertz, modified Gompertz, and Lindstrom equations as follows:

(8) $$\mathrm{Gompertz}:\;N=a\;\cdot \;\exp\left(-\exp\left(b-c\cdot t\right)\right)\;\mathrm{modified}\;\mathrm{Gompertz}:\;\ln\;N=A\;\cdot \;\exp\;\left(-\exp\left(\frac{\mu \cdot 2.718}{A}\cdot \left(\lambda -t\right)\right)+1\right)\;\mathrm{Lindstrom}:\;N=\frac{K}{\left(1+\exp(-r\cdot \left(t-s)\right)\right)}$$

where *t* = time (h); *a*, *b*, *c* = proportional coefficients; *A*, *K* = maximum value of *N*; *µ*, *r* = maximum rate of N increase (1 per h); *λ* = lag time (h); *s* = time (h) to midpoint of exponential portion of curve (that is when *N* = *K/*2). Although developed for OD data, these functions appear suitable also to model population density (CFU per ml) and the production of some metabolites, which could experience a sigmoidal trend (for example ethanol or glycerol).

### Quality and Higher Alcohols

The synthesis of higher alcohols and esters during fermentation makes an important contribution to wine quality, and the control of the production of these volatile compounds is one of the major ways to control the organoleptic characteristics of wine [11]. Morakul et al. [11] proposed a mathematical model for the prediction of the partition coefficient *k_i_*, which is the ratio between the mass concentration of the compound in the gas phase (C_i_^gas^ in mg/L) and that in the liquid phase (C_i_^liq^ in mg/L) at equilibrium. The benefits of predicting *k_i_* include, among others, the calculation of the total production of the volatile compounds from a single measure (concentration in the gas or in the liquid phase). The ability to calculate the total production and differentiate between the amounts remaining in the liquid and those lost in the CO_2_ is a major factor in improving our understanding of yeast metabolism and optimising fermentation control.

According to the authors, the proposed model allowed estimation of the *k_i_* with less than 10% error, except for ethyl acetate. For some molecules, such as isobutanol, the losses in the gas are negligible, but for more volatile compounds, in particular esters, such losses can represent a very significant proportion of the total production. Minimising these losses by optimising the fermentation control, particularly the temperature profile, is a significant challenge. The objective is to find the best compromise between fermentation kinetics and aroma production.

## 3. Malolactic Fermentation

Malolactic fermentation (MLF) is an important step that consists of converting L-malic acid into L-lactic acid after alcoholic fermentation [12]. MLF allows wine deacidification and improves flavour complexity and microbiological stability [13]. *Oenococcus oeni* is the main bacterial species carrying out MLF, due to its ability to grow under the harsh conditions present in wine, such as high ethanol content (>13% *v*/*v*), low pH (<3.5), and high sulphite concentration (<50 ppm) [13].

The link between specific growth and specific L-malic acid consumption was evaluated and quantified using a kinetic model by Fahimi et al. [14]. The following equation was proposed:

(9) $$\nu =k_{i}\;\mu \;\;\frac{\left[mal\right]}{\left[k_{mal}\right]+\left[mal\right]}\;$$

where *ν* is the specific L-malic acid consumption rate; *µ* is the specific growth rate; [*mal*] is the L-malic acid concentration; *k_i_* is a parameter representing the coefficient of proportionality between *ν* and *μ*; it informs us about the intrinsic capacity of the cells of a strain to consume L-malic acid. *k_mal_* is a substrate limitation parameter (expressed in g/L)—a low value of *k_mal_* means that the bacteria can grow with a low concentration of L-malic acid in the medium; conversely, a high value of *k_mal_* means the bacteria require a high minimum threshold of L-malic acid concentration to ensure their growth via the malate metabolism.

Knowing the initial concentration of L-malic acid in the culture medium and after determination of *k_i_* and *k_mal_*, the modelled specific L-malic acid consumption rate (*ν_m_*) was used to calculate the concentration of L-malic acid corresponding to the experimental biomass (*X_exp_*) measured during bacterial growth, as follows:

(10) $$\nu_{m}=\frac{1}{X_{exp}}\;\;\frac{\Delta \;\left[mal\right]}{\Delta t}$$

It was thus possible to deduce the L-malic acid concentration for different time intervals during the MLF and compare it to experimental values. According to the authors, the proposed model seems to be suitable for predicting the concentration of L-malic acid consumed by *O. oeni* bacteria from the values of biomass concentration versus time.

Although selected *O. oeni* strains are available for winemakers, the MLF is not always successful, sometimes because of the indigenous presence of other strains. Brandam et al. [12] proposed a mathematical model to quantify and compare the link between the specific growth rate versus the specific L-malic acid consumption rate for each of the strains. In mixed cultures, the consumption of L-malic acid can be calculated by using the experimental data of the biomass concentrations of each of the two strains according to the following equation:

(11) $$\frac{d\left[mal\right]}{dt}=X_{s1,mixed}\;\;k_{1}\;\mu_{1}\;\;\frac{\left[mal\right]}{\left[mal\right]+k_{mal1}}+X_{s2,mixed\;}\;k_{2}\;\;\mu_{2}\;\;\frac{\left[mal\right]}{\left[mal\right]+k_{mal2}}$$

where *s*1 is strain 1, and *s*2 is strain 2. This predicted consumption in the mixed culture of a pair of bacteria was then compared with their experimental consumption, to reveal if there is an effect of the presence of the other bacterium strain on the intrinsic capacity of the cells of a strain to consume L-malic acid. According to the authors, a large variability between the 10 pairs of *O. oeni* studied was observed. The pairs could be classified into three kinds of interaction based on growth curves analysis: (1) negative reciprocal interaction of both strain growth, (2) interaction that negatively affects the growth of one strain and positively the growth of the other, and (3) positive interaction.

This model could have a strong practical implication because it could help users to understand and know a priori if MLF starter could prevail on the indigenous microbiota.

## 4. Response Surface Approach

The response surface (RS) approach is a powerful tool in food microbiology; it is named RS because of the main output of this technique, that is, three-dimensional plots showing the effects of two independent variables (labelled as X and Y) on the dependent variable (Z) [15,16]. Apart from this output, the goal of RS is to highlight the effects of more dependent variables (from 2 to 8/9) on a dependent variable (viable count, lag phase, growth, death rate, etc.). The approach comprises three main steps: (1) planning the experiment; (2) performing the experiment; (3) modelling results and building an ‘a posteriori’ model. For the first step, the planning of the experiment is performed through a design of experiment (DoE) methodology, which helps researchers to combine different variables, reducing at the same time the number of experiments without affecting the robustness of the results. There are several kinds of DoE, depending on the aim of the study and type of models (third step of RS) to build. The second step concerns performing the experiments in the lab, and the third relies on conducting statistics; generally, modelling is based on a multiple regression (a stepwise approach through a backward or a forward methodology) to build a polynomial equation as follows:

(12) $$y=B_{0}+\sum B_{i}x_{i}+\sum B_{ii}x_{i}{}^{2}+\sum B_{ij}x_{i}x_{j}$$

where ‘*y*’ is the modelled dependent variable (lag phase); *B_i_*, *B_i_*_i_, and *B_ij_* are the coefficients of the model, associated with independent variables; the term ‘*x_i_*’ highlights the individual effect of predictor; the symbols ‘*x_i_*^2^’ and ‘*x_i_x*_j_’ indicate the quadratic and interactive effects. The second output of RS is the set of three-dimensional plots showing the effects of independent variables on the dependent one, as reported above [15].

A quadratic RS methodology was adopted by D’Amato et al. [17]; they used as input factors temperature, sugar, and ammonium concentrations, while the dependent variables were population density and weight loss. The correlation of cell count vs. nitrogen was negative, as the highest survival of the yeast was found at low concentrations of ammonium; in addition, the equations stressed the importance of the interactive factors.

Most of the commercial yeasts belong to the *S. cerevisiae* species; however, other yeasts such as non-*Saccharomyces* species have received great attention in the winemaking industry because of their influences on wine flavour, as well as ethanol, glycerol, and acetic acid production [18].

Arroyo-López et al. [16] studied the effects of temperature, pH, and sugar concentration on the growth of *S. cerevisiae*, *S. kudriavzevii,* and the hybrid strain *S. cerevisiae* × *S. kudriavzevii*. By means of RS methodology, the authors built polynomial equations for the maximum specific growth rate (*μ_max_*) of yeasts. As result, the exponential phase is started without any lag phase and reaches the growth asymptote in a very short time.

Ale et al. [19] used the RS methodology and an optimisation approach to improve the performance of mixed cultures (i.e., *S. cerevisiae* mc_2_, *Kloeckera apiculata* mF, and *Oenococcus oeni* X_2_L) for wine fermentations; the effects of temperature, pH, and SO_2_ on growth and glycerol production were observed. According to the authors, 26 °C, 60.24 mg/L SO_2,_ and pH 5.5 were the optimal conditions for glycerol and organic acid synthesis compatible with wine quality.

## 5. Inactivation of Spoiling Yeasts

During winemaking, there are also yeasts with a negative role or a spoiling effect, including the species *Zygosaccharomyces bailii*, *Schizosaccharomyces pombe*, *Saccharomycodes ludwigii*, and *Brettanomyces bruxellensis*, and even the fermenting species *S. cerevisiae* [20].

Aguilar-Uscanga et al. [21] used a nonstructured Monod type, modified and proposed in Han and Levenspiel [22], to evaluate the effect of glucose concentrations on the growth by *B. bruxellensis* yeast strain. The model was built upon the following assumptions:

**Assumption** **1.** *Oxygen is the limiting substrate, while ethanol exerts a negative feedback effect; these two variables contribute to dividing yeast growth cycle into three phases, as follows:*

*(a)*  *Phase 1 (42 h): the medium is aerobic because oxygen is at relevant concentrations;*
*(b)*  *Phase 2 (from 42 h to the end of fermentation): the medium is anaerobic, and the concentration of ethanol gradually increases;*
*(c)*  *Phase 3: ethanol exerts a secondary inhibiting effect, independent from its high concentration in the medium.*

**Assumption** **2.** *There are inhibition phenomena depending on the substrate also in the first phase of the fermentation.*

**Assumption** **3.** *The kinetic constant of oxygen (K_m,o_) is independent of sugar concentration.*

These assumptions were used to write a set of three main equations, as follows:

Phase 1:

(13) $$\mu_{1}=\mu_{max}\left(\frac{C_{L}}{C_{L}+K_{m,o}}\right){\left(1-\frac{S}{S^{*}}\right)}^{n}$$

In this equation *C_L_* > 0 (aerobic conditions) and *n* > 0. For phase 2, when (*C_L_* = 0), the specific growth rate was determined by

(14) $$\frac{dC_{L}}{dt}=K_{La}\left(C^{*}-C_{L}\right)-\frac{\mu x}{Y_{O}}=0$$

where

(15) $$\mu_{2}=\frac{Y_{O}K_{La}C^{*}}{x}$$

For phase 3, when *P* = *P_c_* (critical production concentration),

(16) $$\mu_{3}=\mu_{max}\left(1-\frac{P_{c}-\alpha \mu_{max^{X}}}{\alpha \mu_{max^{X}}}\right)$$

According to the authors, the model well simulated the batch kinetics observed in all cases. At a glucose concentration of 50–138 g/L, the ethanol and biomass production were 24, 59, and 6.3, 11.4 g/L, respectively; an increase in glucose concentration to 165 g/L led to a drastic decrease in product formation and substrate utilisation.

The symbols are *μ_max_*, maximal specific growth rate; *Y*_0_, biomass on oxygen yield coefficient; *K_m,o_*, Monod saturation constant for oxygen as limiting substrate; S*, substrate inhibitory concentration in the reactor; *K_La_*, oxygen transfer reactor coefficient; C*, gas–liquid equilibrium oxygen concentration; *n*, the number of parameters estimated; *P_c_*, ethanol concentration critic in the reactor; α, ethanol on biomass yield coefficient.

In another study, Medawar et al. [23] proposed a model to evaluate the risk of *Brettanomyces* sp. growth in an alcoholic medium. The general equation was as follows:

(17) $$\lambda =\lambda_{0}{\left(1-\frac{\mathrm{EtOH}}{{\mathrm{EtOH}}^{*}}\right)}^{n}$$

This model is based on the concept of a critical ethanol concentration (EtOH*) and a curve with a hyperbolic shape; *λ*_0_ is the lag phase without ethanol. The best fit of the data according to the authors was found with *n* = −1.

Chandra et al. [20] used RM methodology (central composite rotatable designs) to study the effect of sulphur dioxide, ethanol, and glucose, on the growth of *Z. bailii*, *Sc. pombe*, *Sa. ludwigii*, and *S. cerevisiae*. They used a classical approach based on polynomial equations with linear, quadratic, and, two-way interactions, and surface response plots and focused on the cell density at selected time points (2, 5, 15, and 30 days). The result is a time-by-time analysis of the systems as a set of images used to gain a general overview. Despite the limits of this static approach, used also by other authors and for other purposes, Chandra et al. [20] were able to collect some important data in the light of preventing the growth of spoiling yeasts; for example, they found that *S. ludwigii* was the most resistant species growing in the following conditions (ethanol/sulphur dioxide): 15%/20 mg/L, 14%/32 mg/L, and 12.5%/40 mg/L. The effect of SO_2_ was reversible as yeasts adapted for a prolonged incubation, as shown by *S. ludwigii* and *S. cerevisiae*. Finally, the most important practical result of these authors was the finding that sugar levels commonly used in wine to sweeten the mouthfeel do not increase wine susceptibility to spoilage yeasts.

## 6. Toxin Production and Removal

Mycotoxins are natural secondary metabolites produced by fungi with strong evidence of correlation with cancer and other diseases; among these compounds, the most important moiety for wine microbiology is ochratoxin A (OTA). It is produced by several fungal species including *Penicillium* section *Circumdati* and *Aspergillus* section *Nigri* and has nephrotoxic, immunosuppressive, teratogenic, and carcinogenic effects on animals and humans. In winemaking, *Aspergillus carbonarius* was the fungus most involved in the cases of contamination [24].

Tassou et al. [24] developed a logistic regression model to predict growth and OTA production by *A. carbonarius* as a function of temperature and water activity (aw).

The model employed was as follows:

(18) $$\log it\left(P\right)=\ln\left[\frac{P}{1-P}\right]=a_{0}+a_{1}t+a_{2}T\;+a_{3}a_{w}+a_{4}t^{2}+a_{5}T^{2}+a_{6}a^{2}{}_{w}\;+a_{7}tT+a_{8}ta_{w}+a_{9}Ta_{w},$$

where *P* is the probability of growth (in the range of 0–1), a_i_ is the regression coefficients of the various factors (from a_1_ to a_7_), and *t* is the time. The model was robust and showed a degree of agreement between predictions and observations > 99%. However, it is a posteriori approach, useful to model experimental data and to understand the phenomenon, but it has a limited significance for predictive purposes.

An ‘a posteriori method’ was also proposed by Ioannidis et al. [25], who studied the effect of temperature (15–38 °C), aw (0.88–0.98), and sodium metabisulphite (NaMBS) concentration (0–200 mg/L) on the growth and OTA production by *A. carbonarius*.

According to the authors, changes in colony diameter were plotted against time; the maximum growth rate (*m_max_*) and lag phase duration were obtained by fitting the data to the primary model of Baranyi and Roberts [26] modified as follows [27]:

(19) $$D\left(t\right)=\mu_{max}A-\log\left\{1+\frac{\left[\exp\left(\mu_{max}A\right)-1\right]}{\exp\left(D_{max}\right)}\right\}$$

as well as

(20) $$A=t+\frac{1}{\mu_{max}}\log\left[\exp\left(-\mu_{max}t\right)+\exp\left(-\mu_{max}\lambda \right)-\exp\left(-\mu_{max}t-\mu_{max}\lambda \right)\right]$$

where *D*(*t*) is the changes in colony diameter versus time (mm), *μ_max_* is the maximum growth rate (mm h^−1^), and *λ* is the lag phase duration (h); further, *μ_max_* and *λ* were fitted to the secondary cardinal model with inflection (CMI) [28], to define the effect of temperature.

This approach (surface response approach and a posteriori methodology) was used to model the ability of yeasts to act as adsorbing tools, to remove OTA during and after the fermentation [29,30].

Petruzzi et al. [30] used a completely randomised design and studied the effect of time, temperature, sugar content, and addition of diammonium phosphate (DAP) on OTA removal by two strains of *S. cerevisiae* [30]. Temperature (25 and 30 °C), sugar (200 and 250 g/L) and DAP (0 and 300 mg/L) were used as independent variables or factors; each of them was set at two different levels (‘−1′ and ‘+1′, respectively, the minimum and the maximum levels of each variable). The analysis was performed through the option DoE/2^(k–p)^ standard designs/two-way interactions; OTA reductions after 3 and 10 days were used as dependent variables. According to the contour plots, OTA reduction was maximum at 30 °C, sugar at 250 g/L, and DAP at 300 mg/L. The equation was cast as a polynomial function, as reported for the RS approach.

Finally, a different modelling approach with possibilities of a priori applications (predictive purposes) was used by Battilani and Camardo Leggieri [31] with a nonconventional procedure for PM. They studied *A. carbonarius* during the whole life cycle of grape and pointed out some primary variables (overwinter inoculum of fungus, spores on berries, germinated spores’ growth on berries, infected berries, colonised berries) and intermediate variables (berry condition and growth phase of berries). The model is a relational diagram combining a set of rate values (dispersal, germination, growth, infection, colonisation, and OTA production) and some weather parameters (temperature, humidity, rainfall, water activity, pest, and disease); the rates can be imposed by users from 0 (lack) and 1 (presence or high values). The output of this diagram is the probability of contamination of berries (risk) as a tool to predict the probability of contamination of grapes.

## 7. Perspectives of PM for Wine Microbiology

Although the use of PM has been proposed since the beginning of the 2000s, for wine microbiology, there are some unexplored fields for effective use for oenological purposes. In this paper, our aim was to show possible applications and potentialities and offer an overview.

Future advances in wine PM are related to the following aspects:

1. Stochastic approaches for modelling individual growth or death kinetics;
2. Efficient use of molecular microbiology;
3. Computational microbiology and bioinformatics progress;
4. Further studies for a priori and predictive models;
5. Exploring the possibility of building a comprehensive/holistic model able to connect data on the dynamics of populations (yeasts, lactic acid bacteria, spoiling microorganisms) and the amounts of some compounds acting on the sensory profile of wines. At present, this topic is lacking.

These directions could be of interest for wine microbiology, but the knowledge of PM models for global wine industry applications is a major challenge. The presented review in this paper is a tool that may help to decide on the right models and their practical use. We suggest an interdisciplinary collaboration of microbiologists and mathematicians, technologists, computer scientists, and statisticians in order to accelerate PM innovation.

## Data Availability

Not applicable.
